# Effects of Acceptance and Commitment Therapy (ACT) on Mental Health and Resiliency of Migrant Live-in Caregivers in Canada: Pilot Randomized Wait List Controlled Trial

**DOI:** 10.2196/32136

**Published:** 2022-01-27

**Authors:** Mandana Vahabi, Josephine Pui-Hing Wong, Masoomeh Moosapoor, Abdolreza Akbarian, Kenneth Fung

**Affiliations:** 1 Daphne Cockwell School of Nursing Ryerson University Toronto, ON Canada; 2 Toronto Western Hospital University Health Network Toronto, ON Canada

**Keywords:** migrant live-in caregiver, women, mental health, acceptance and commitment therapy, depression, anxiety, stress, psychological inflexibility, resilience, social identity

## Abstract

**Background:**

Temporary migrant live-in caregivers constitute a vulnerable stream of temporary foreign workers in Canada. This is because the majority are racialized women from the Global South, the gendered nature of caregiving work has historically been undervalued, and their working and living spheres are intertwined which makes application of labor laws and surveillance almost impossible. Their invisible position in the fabric of Canadian society along with their precarious employment and immigration status place their mental health at jeopardy. There is a paucity of research about psychological support for this population.

**Objective:**

Our pilot study Women Empowerment—Caregiver Acceptance and Resilience E-Learning (WE2CARE) aimed to assess the efficacy of a 6-week online delivery of a psychological intervention based on acceptance and commitment therapy (ACT) in reducing psychological distress and promoting resiliency among live-in care givers in the Greater Toronto Area.

**Methods:**

A pilot randomized wait list controlled design was used. Participants were recruited by two community peer champions working with community health organizations serving migrant live-in caregivers. A total of 36 participants were recruited and randomly assigned to the intervention and wait list control groups; 7 dropped out of the study due to competing life priorities. Standardized self-reported surveys were administered online pre-, post-, and 6-week postintervention to assess mental distress (DASS-21), psychological flexibility (AAQ-2), mindfulness (CAMS-R), and Multi-System Model of Resilience (MSMR-I). Independent and dependent *t* tests were used to compare study outcomes at pre, post, and 6-week follow-up across and within both arms of the study. Linear mixed effects models were created for each outcome of interest from baseline to postintervention among intervention and control participants. Self-reported impact of the WE2CARE intervention was examined using independent *t* tests across the study arms.

**Results:**

Average age of participants was 38 years. Many were born in the Philippines (23/29, 79%). The data on the impact of the psychological intervention showed a lower level of depression, anxiety, and stress among the intervention group compared with the control. However, the differences were not significant due to small sample size and COVID-19 crisis (6.94 vs 9.50, *P*=.54; 6.94 vs 10.83, *P*=.20; 7.76 vs 10.33, *P*=.44, respectively). There was a significant improvement in mindful qualities and external resilience, particularly in life satisfaction and accessible support among the intervention group (37.18 vs 32.92, t_22_=2.35, *P*=.03; 20.29 vs 16.5, t_21_=2.98, *P*=.007; 8.47 vs 6.75, t_14_=2.41, *P*=.03; 7.59 vs 5.33, t_16_=.008, respectively).

**Conclusions:**

WE2CARE is among the first studies exploring the efficacy of online delivery of ACT in addressing mental health challenges among live-in caregivers. While there are increased web-based ACT interventions, few use group videoconferencing to promote peer connection and mutual support. WE2CARE showed promising results in reducing psychological distress and promoting mindfulness and resiliency. The intervention highly motivated participants to engage collectively in building social support networks.

**International Registered Report Identifier (IRRID):**

RR2-10.2196/preprints.31211

## Introduction

The Caregiver Program, previously known as the Live-in Caregiver Program, is a stream of the Canadian Temporary Foreign Workers Program, which engages workers in home caregiving [1,2]. Workers who enter the country under this program constitute an underrepresented and vulnerable stream of temporary foreign workers in Canada since more than 90% of workers are women of color from the Global South; their employment falls in the purview of feminized work that historically and globally has been devalued, dehumanized, and underpaid; and the location of their services situated in the private sphere derails the application of labor laws, government surveillance, and unionization [3-5].

Under the Live-in Caregiver Program, migrant caregivers were restricted to work and live only for the employers stated on their work permits while providing care to their employers’ children, seniors, or disabled family members. Despite Caregiver Program reforms in November 2014 which provided caregivers with the option of living outside of their employers’ homes, most caregivers continued to live with their employer due to low wages, inability to afford independent housing, and precarious work permits [6].

Most caregivers are racialized women from lower- or middle-income countries, with the majority coming from the Philippines and a growing number from Haiti, Africa, Latin America, and Asia [1,3,7]. They are often the sole provider of income for their families back home, and their need to send regular remittances forces them to accept a submissive and discriminatory position, relinquish their basic human rights, and submit to precarious employment, financial exploitation (low wages, long working hours without compensation), emotional and physical abuse, and having little to no access to social or health care services despite paying into these programs [3,8,9]. Furthermore, the possibility of applying for permanent residency for not only themselves but also their immediate family members after completing 24 months of service often acts as an impetus for caregivers’ acceptance of their dire living and working conditions in Canada. Considering that family separation is one of the requirements for caregivers’ work permits in Canada under both the Live-in Caregiver Program and Caregiver Program, securing permanent residency for caregivers symbolizes the hope for family reunification. It is noteworthy that the process of gaining permanent residency for this group can take from 4 to 10 years. While Caregiver Program reforms in November 2014 have included a commitment to reduce processing times for permanent residency of caregivers [10,11], the processing time until 2016 remained at a minimum average of 6 years.

Most studies with temporary migrant workers explored their vulnerability in the areas of occupational injury and hazard, sexual and reproductive health, and chronic and infectious diseases like HIV/AIDS [12-20]. These vulnerabilities have been reported to be associated to temporary migrant workers’ working and living conditions such as discrimination, precarious work permit and immigration status, stigma, limited social support, and fear of job loss (12,14,17-20].

Although live-in caregivers face a multitude of challenges in the host countries, there is limited research related to the impact of these challenges on their mental health. The few studies that have explored this issue found a high level of psychological distress, feeling of alienation and loneliness, and limited social support [9,21]. Multiple barriers to accessing mental health services were also reported including long working hours, fear of job loss and deportation, and limited knowledge about available mental health resources. Considering these barriers, offering online psychosocial support programs can be an effective strategy in reducing temporary migrant live-in caregivers’ psychological distress and promoting their mental well-being.

Acceptance and commitment therapy (ACT) is a cognitive behavioral approach that promotes psychological flexibility. Psychological flexibility is the aptitude of adjusting to any situational demand which in turn allows for living a meaningful life. ACT targets psychological flexibility through advancing 6 core skills: acceptance (experiences of both pleasant and unpleasant thoughts, emotions, and feelings instead of trying to avoid or control them), defusion (stepping back and observing thoughts as thoughts), contact with the present moment (consciously engaging in any moment and being mindful of thoughts, feelings, and actions), self-as-context (awareness and self-perspective), values (being clear about what matters), and committed action (taking actions that are guided by one’s values) [22,23]. All these processes can be viewed as efforts directed at relinquishing antecedent stimulus control that exist due to verbal conditioning. In other words, ACT does not focus on reducing clinical symptoms but rather aims at altering their behavioral impact. This is done through deconstructing the individual experience in the context of personal values and acceptance of both negative and positive components of experience. Acceptance and mindfulness are core processes of psychological flexibility [24,25].

A significant body of literature has provided support for the efficacy of ACT in promoting well-being and reducing psychological distress among both clinical and nonclinical populations [26-29]. Furthermore, the internet-based delivery of ACT has been reported to be promising in managing anxiety, depression, chronic pain, and distress related to trauma and promoting mental wellness and psychological flexibility [30-32].

Engaging migrant live-in caregivers in learning ACT skills can support their mental health by decreasing distress through strategies like mindfulness, defusion from negative thoughts, and increasing committed action consistent with the value of self-care (eg, engaging in culturally syntonic activities like singing or praying and building social support networks). Given that live-in caregivers work long hours and have extremely limited free time and restricted social support, a web-based approach is the best-suited medium for the delivery of self-help psychological treatment in this population. Earlier studies with this population reported a preference for online health resources among this population due to their precarious work permit and fear of repatriation—being sick or seeking medical care may put their livelihood in jeopardy [9,21]. This approach offers flexible access while promoting virtual social connection. To our knowledge there is a paucity of research about the effectiveness and suitability of an internet-delivered psychological intervention based on ACT for this population.

Our pilot study, Women Empowerment—Caregiver Acceptance and Resilience E-Learning (WE2CARE), aimed to address this gap by exploring the efficacy of a 6-week online psychological intervention based on ACT for temporary migrant live-in caregivers.

## Methods

### Theoretical Framework

Our study was guided by the population health promotion framework, which is grounded in the principles of social justice and equity [33-35]. This framework acknowledges that health disparities are the outcomes of myriad social determinants including access to physical, psychological, social, and financial security. In this study, we recognized that the life circumstances of live-in caregivers are shaped by systemic issues in both Canada and their countries of origin. As upstream strategies (policy reform and resource redistribution) will take years to implement and achieve, it was critical to implement midstream (supportive environment, community engagement) and downstream (individual coping, self-care) strategies to address the urgent mental health needs of live-in caregivers. To address the latter, we applied culturally safe and adult learning principles.

### Design

A pilot community-based mixed methods study was used to examine the feasibility and efficacy of WE2CARE in reducing psychological distress (depression, anxiety, and stress) and promoting committed actions of self-care and identify perceived barriers and facilitators to using WE2CARE. In this paper, we report only on the findings from the quantitative component of the study, which consisted of a randomized controlled wait list design and explored the feasibility and efficacy of the intervention.

### Participants and Recruitment Strategies

**Figure 1:**
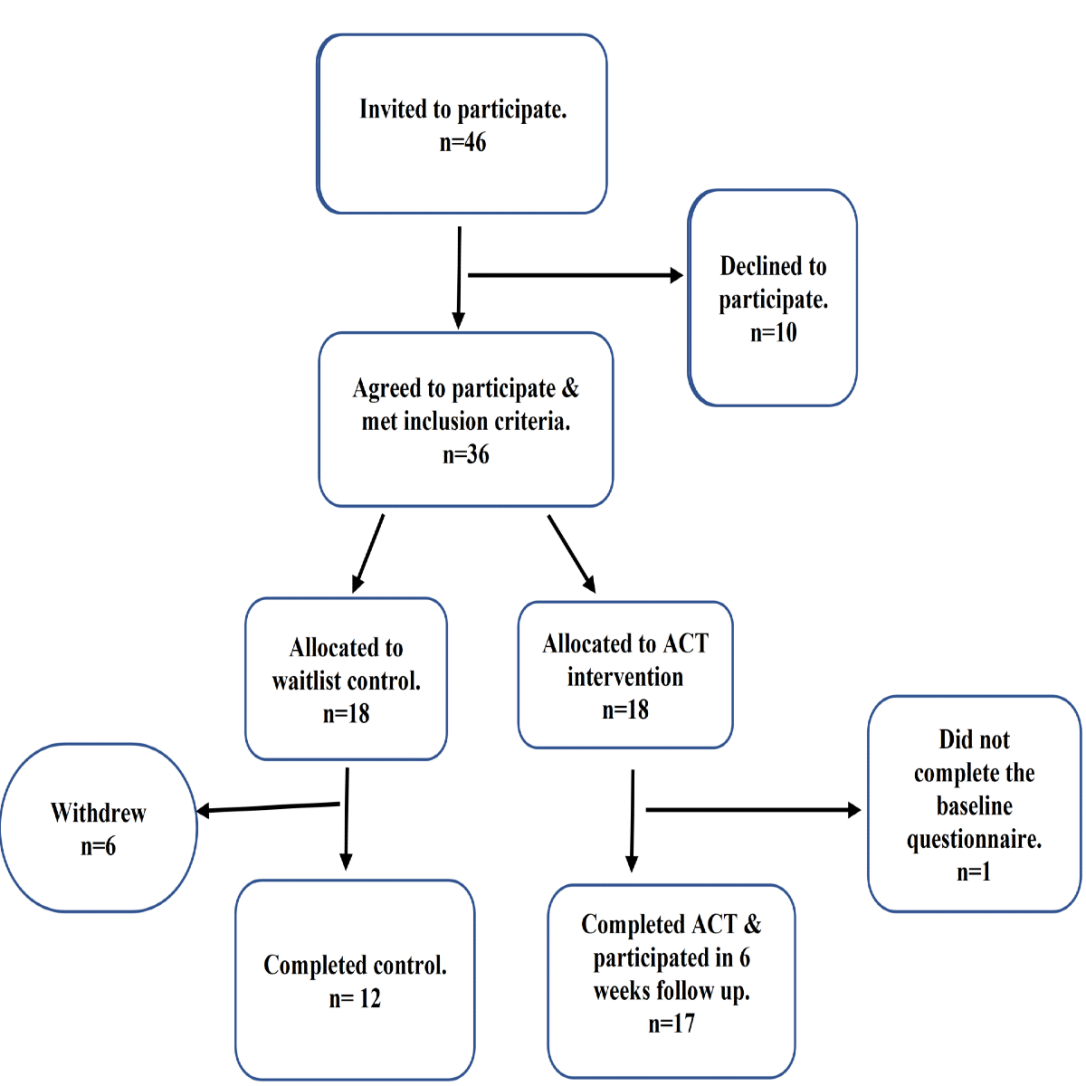
Flowchart of WE2CARE study participants.

The study protocol received ethical approval from the research ethics review boards at the affiliated universities. Those included Ryerson University (REB 2019-036) and University of Toronto (RIS37623). A total of 36 participants were recruited by 2 community champions (trusted members of live-in caregivers’ community) and snowball technique. Eligible participants met the study inclusion criteria: (1) self-identified as female aged 18 years or older, (2) residing in the Greater Toronto Area, (3) working on a temporary work permit as a live-in caregiver, (4) able to speak and read English, (5) had internet access, and (6) able to take part in the 6-week intervention. They were randomly divided into the intervention arm and wait list control arm using a random number generator. Of 18 participants in the intervention group, 1 did not complete the baseline questionnaire, while 6 of 18 participants in the control group withdrew from the study due to other competing life priorities. The total number of participants who completed the pilot study was 29, with 17 in the intervention group and 12 in the wait list control group. The intervention group was further divided into 2 cohorts, 9 and 8 participants each, to enhance participation. After those in the intervention group received the 6-week online intervention, it was offered to the control group to respect the ethical principle of beneficence (Figure 1). All participants were compensated for their participation in the study and the cost of internet use.

### WE2CARE Intervention

The WE2CARE intervention consisted of 6 e-learning modules that explained the ACT processes. Each week, participants were invited to complete an online self-directed, interactive experiential session on ACT strategies (approximately 1 hour to complete) and attend a 1.5-hour online live videoconference. The videoconference was designed to support participants in applying ACT strategies in their everyday life and consisted of a group discussion, peer sharing, and question and answers. The videoconference was facilitated by 2 of our research team members with extensive knowledge and experience in ACT. Prior to each videoconferencing, which took place on mutually agreed weekdays in the evening, participants were given a week to complete their assigned weekly module. Details of the study protocol have been reported elsewhere [36].

### Data Collection and Measures

Data were captured through self-report instruments administered online at pre-, post-, and 6-week postintervention. The preintervention survey included sociodemographic and health-related questions that were identified as important in previous research with temporary migrant workers [14,16]. The standardized scales administered at pre-, post-, and 6-week follow-up included: (1) Depression, Anxiety and Stress Scale (DASS-21)—a set of 3 self-report scales (21 items) designed to measure the emotional states of depression (DASS-D), anxiety (DASS-A) and stress (DASS-S); Cronbach alpha values of 0.81, 0.89 and 0.78 for the subscales of depressive, anxiety and stress respectively; (2) Acceptance and Action Questionnaire–2 (AAQ-2)—a 7-item scale designed to measure the impact of ACT core process conceptualized as psychological flexibility; (3) Cognitive and Affective Mindfulness Scale (CAMS-R)—a 12-item measure designed to capture a broad conceptualization of mindfulness not specific to any particular type of meditation training; and the (4) Multi-System Model of Resilience (MSMR-I), consisting of 3 subscales: internal resilience (MSMR-IR), coping pursuits (MSMR-CP), and external resilience (MSMR-ER). Each subscale contains 9 self-reported items and indicates where the barriers to one’s resilience lie. These scales have shown good psychometric properties including internal consistency, test-retest reliability, and validity. For instance, the depressive, anxiety, and stress subscales in DASS have been have found to have excellent Cronbach alpha values of .81, .89, and .78, respectively. AAQ-2 was reported to have good internal consistency (α=.88) and good test retest reliability over 3 and 12 months at .81 and .79, respectively. CAMS-R was reported to have good Cronbach alpha (.67) and good convergent validity that is supported by its negative relationship to the DASS-21 is negatively correlated to DASS (–.28). MSMR-I also showed excellent internal consistency with Cronbach alpha of .90 and high test-retest reliability .84 [37-43].

### Data Analyses

Descriptive statistics were used to summarize participants’ sociodemographic and self-reported health characteristics. Fisher exact tests were used to compare participant characteristics by study arm allocation. Both independent and dependent *t* tests were used to compare study outcomes pre, post, and 6-week follow-up across and within both arms of the study. Linear mixed effects models were created for each outcome of interest from baseline to postintervention among intervention and control participants. Self-reported impact of the WE2CARE intervention was examined using independent *t* tests across the study arms. Data were entered and analyzed using the SPSS Statistics (version 27, IBM Corp). A threshold of .05 was used to determine the level of significance for all *P* values.

## Results

### Sociodemographic and Self-reported Health Characteristics

Average age of participants was 38 years. Many participants were born in the Philippines (23/29, 79%) and have lived in Canada for more than 24 months (17/29, 59%). Most participants were married (12/29, 41%) and had children (18/29, 62%), but none of their children were in Canada. Most had college or university degree (19/29, 66%) and rated their English literacy as being good (19/29, 66%). Nearly 90% (26/29) of participants were temporary workers with a valid visa and work permit; 83% (24/29) worked as live-in caregivers, 79% (23/29) worked full-time, and 62% (18/29) worked 35 to 44 hours per week. Among those who reported their income, 59% (13/22) earned less than CAD $24,999 (US $19,441) after taxes. Although most (23/29, 79%) participants did not share their sleeping area or bedroom with others, for those who did share, they shared their sleeping area with, on average, 2 other people. Lack of privacy was a common concern raised by participant who were concerned about their accommodation.

A total of 7% (2/29) of participants rated their health at the time of entry to Canada as poor or fair compared with other people their age. However, this proportion was considerably increased (8/29, 28%) after arrival to Canada. Regarding satisfaction with life, 10% (3/29) were dissatisfied or very dissatisfied and 24% (7/29) were neither satisfied nor dissatisfied. The mean self-rated level of stress was more than 50 on a scale of 0 to 100 (0 being no stress and 100 being extremely stressful). About a fifth (6/29, 21%) of the participants rated their mental health as fair or poor compared with other people of their own age. More than half (15/29, 52%) of the participants were dissatisfied with their quality of sleep and reported having difficulty falling sleep, staying asleep, or getting sufficient hours of sleep. Overall, participants were relatively healthy before and after coming to Canada although they felt their overall physical and mental health were gradually deteriorating. Only a few people had been diagnosed with high blood pressure, cancer, intestinal/stomach problems, or depression since arriving in Canada.

More than 50% (17/29) of participants had been tested for HIV (a required medical test for entry to Canada), and of those, 41% (7/29) stated that their test was done sometime from 2018 to 2019. Interestingly, the rate of screening for sexually transmitted infections appeared to be quite low among this population. Most (20/29, 69%) participants stated that they have never been tested for human papillomavirus, a common cause of cervical cancer, or any other sexually transmitted infection (21/29, 72%).

Participants were asked about their use of health care services since coming to Canada. A total of 28% (8/29) did not use health care services while the rest did. A wide variety of reasons were given; the most common reason (8/21, 38%) was annual checkup followed by job requirement for flu vaccination (3/21, 14%).

There were no differences between the intervention and control groups on demographic variables and self-reported health characteristics as can be seen in Table 1.

**Table 1:** Sociodemographic and clinical characteristics of study participants.

					Overall (n=29)			Intervention (n=17)			Control (n=12)		*P* value^a^
**Demographic characteristics**													
	Age (years), mean (SD)			38.38 (6.62)			38.41 (7.11)			38.33 (6.18)			.98
	**Country of birth, n (%)**												.92
		Philippines	23 (79)			13 (77)			10 (83)			—^b^	
		Other	6 (21)			4 (24)			2 (17)			—	
	**Time lived in Canada, n (%)**												>.99
		Less than 12 months	3 (10)			2 (12)			1 (8)			—	
		12 months or more	26 (90)			15 (88)			11 (92)			—	
	**Immigration status, n (%)**												.70
		Refugee applicant	1 (3)			0 (0)			1 (8)			—	
		No status	2 (7)			1 (6)			1 (8)			—	
		Temporary foreign worker with a valid visa and work permit	26 (90)			16 (94)			10 (83)			—	
	**Marital status, n (%)**												.68
		Divorced	2 (7)			2 (12)			0 (0)			—	
		Married	12 (41)			6 (35)			6 (50)			—	
		Separated	1 (3)			1 (6)			0 (0)			—	
		Single (never married)	11 (38)			7 (41)			4 (33)			—	
		Widowed	3 (10)			1 (6)			2 (17)			—	
	**Has children, n (%)**												—
		Yes	18 (62)			9 (53)			9 (75)			.27	
	**Has children in Canada, n (%)**												—
		No	18 (100)			9 (100)			9 (100)			>.99	
	**English literacy, n (%)**												.09
		Poor	1 (3)			1 (6)			0 (0)			—	
		Fair	5 (17)			1 (6)			4 (33)			—	
		Good	19 (66)			11 (65)			8 (67)			—	
		Very good/excellent	4 (14)			4 (24)			0 (0)			—	
	**Education, n (%)**												.35
		Less than high school (grade 8 or less)	1 (3)			0 (0)			1 (8)			—	
		High school (grade 12) or equivalent	7 (24)			3 (18)			4 (33)			—	
		College (eg, diploma) or university (eg, BA, BSc)	19 (66)			12 (71)			7 (58)			—	
		Some or completed postgraduation (eg, Master’s, PhD)	2 (7)			2 (12)			0 (0)			—	
	**Employment status, n (%)**												.87
		Full-time (minimum of 35 hours/week)	23 (79)			14 (82)			9 (75)			—	
		Part-time	2 (7)			1 (6)			1 (8)			—	
		Unemployed	3 (10)			1 (6)			2 (17)			—	
		Other	1 (3)			1 (6)			0 (0)			—	
	**Work hours per week, n (%)**												—
		0	3 (10)			1 (6)			2 (17)			—	
		Less than 24	1 (3)			0 (0)			1 (8)			—	
		25-34	1 (3)			1 (6)			0 (0)			—	
		35-44	18 (62)			12 (71)			6 (50)			—	
		45+	6 (21)			3 (17)			3 (25)			—	
	**Current occupation, n (%)**												.69
		Live-in caregiver	24 (83)			15 (88)			9 (75)			—	
		Personal support worker	2 (7)			1 (6)			1 (8)			—	
		Not employed	3 (10)			1 (6)			2 (17)			—	
	**Income after taxes (CAD $), n (%)**												.89
		Less than $24,999	13 (45)			7 (41)			6 (50)			—	
		$25,000-$39,999	9 (31)			6 (35)			3 (25)			—	
		Prefer not to answer	7 (24)			4 (24)			3 (25)			—	
**Health assessment**													
	**Self-rated general health when arrived in Canada, n (%)**												.30
		Poor	0 (0)			0 (0)			0 (0)			—	
		Fair	2 (7)			0 (0)			2 (17)			—	
		Good	11 (38)			7 (41)			4 (33)			—	
		Very good/excellent	16 (55)			10 (59)			6 (50)			—	
	**Self-rated general health since arrival in Canada, n (%)**												.94
		Poor	2 (7)			1 (6)			1 (8)			—	
		Fair	6 (21)			3 (18)			3 (25)			—	
		Good	11 (38)			7 (41)			4 (33)			—	
		Very good/excellent	10 (35)			6 (35)			4 (33)			—	
	**Life satisfaction since arrival in Canada, n (%)**												.95
		Very dissatisfied	1 (3)			1 (6)			0 (0)			—	
		Dissatisfied	2 (7)			1 (6)			1 (8)			—	
		Neither satisfied nor dissatisfied	7 (24)			5 (29)			2 (17)			—	
		Satisfied	17 (59)			9 (53)			8 (67)			—	
		Very satisfied	2 (7)			1 (6)			1 (8)			—	
	Self-rated level of stress since arrival in Canada (0=not at all stressful, 100=extremely stressful), mean (SD)			57.31 (27.47)			53.41 (28.16)			62.83 (26.66)			.37
	**Self-rated general mental health, n (%)**												.50
		Poor	2 (7)			1 (6)			1 (8)			—	
		Fair	4 (14)			1 (6)			3 (25)			—	
		Good	16 (55)			11 (38)			5 (42)			—	
		Very good/excellent	7 (24)			4 (24)			3 (25)			—	
	**Quality of sleep, n (%)**												.71
		Satisfied	14 (48)			9 (53)			5 (42)			—	
		Dissatisfied	15 (52)			8 (47)			7 (58)			—	
	**Diagnosed health conditions since arrival in Canada, n (%)**												.43
		High blood pressure	2 (7)			0 (0)			2 (17)			—	
		Cancer	1 (3)			1 (6)			0 (0)			—	
		Intestinal problems (ulcer, Crohn disease, irritable bowel syndrome)	1 (3)			1 (6)			0 (0)			—	
		Depression or other mood disorders (anxiety)	1 (3)			0 (0)			1 (8)			—	
		STIs^c^	1 (3)			1 (6)			0 (0)			—	
		Others	1 (3)			0 (0)			1 (8)			—	
	**Last tested for HPV^d^, n (%)**												.33
		Before 2013	1 (3)			1 (6)			0 (0)			—	
		2018-2019	4 (14)			3 (18)			1 (8)			—	
		Never	20 (69)			11 (12)			9 (75)			—	
		Not sure	2 (7)			0 (0)			2 (17)			—	
		Prefer not to answer	2 (7)			2 (12)			0 (0)			—	
	**Last tested for STI, n (%)**												—
		2018-2019	3 (10)			3 (18)			0 (0)			.33	
		Never	21 (72)			11 (65)			10 (83)			—	
		Not sure	3 (10)			1 (6)			2 (17)			—	
		Prefer not to answer	2 (7)			2 (12)			0 (0)			.38	
		Last tested for HIV										—	
		Prefer not to answer	3 (10)			2 (12)			1 (8)			—	

^a^Fisher exact test was used to compare count data. *T*-test was used to compare means.

^b^—: not applicable

^c^STI: sexually transmitted infection.

^d^HPV: human papillomavirus.

### Study Outcome Measures: DASS, AAQ-2, CAMS-R, and MSMR-I

Tables 2 and 3 demonstrate the mean score differences within the intervention group before, after and at 6-week follow-up for all the outcome measures. Table 4 shows the mean score differences between the intervention and control groups for all the outcome measures. Tables 5 and 6 show the linear mixed effects regression analyses using the pre- and postassessment on the main outcomes across both groups.

**Figure 2:**
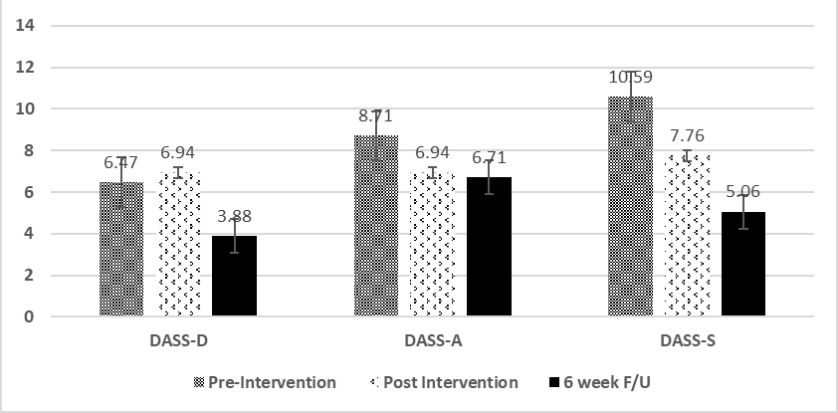
Depression Anxiety Stress Scale (DASS) T1 (Pre) T2 (Post) T3 (6week F/U).

The mean scores for DASS-D, DASS-A, and DASS-S decreased steadily among the intervention arm post and 6-week follow-up: 40% drop in depression level, 23% drop in anxiety level, and 52% drop in stress level (Figure 2). However, these changes were not significant except for the stress level after 6-week follow-up (10.59 vs 5.06, t_16_=2.6, *P*=.02). Furthermore, overall resiliency (MSMR) and external resiliency (MSMR-ER), particularly in the sphere of access to needed support (ie, MSMR accessible support), significantly increased compared to baseline (62.53 vs 65.35, t_16_=–3.33, *P*=.004; 19.65 vs 21.35, t_16_=–2.71, *P*=.02; 6.71 vs 7.88, t_16_=–2.85, *P*=.01, respectively).

**Table 2:** Paired sample *t* test among intervention participants at pre/baseline (T1) and postintervention (T2).

Variable	Baseline (n=17), mean (SD)	Postintervention (n=17), mean (SD)	*t* score	*df*	*P* value
DASS-D^a^	6.47 (10.67)	6.94 (9.83)	–0.26	16	.80
DASS-A^b^	8.71 (9.25)	6.94 (5.57)	0.71	16	.49
DASS-S^c^	10.59 (9.92)	7.76 (6.32)	1.02	16	.32
AAQ-2^d^	18.65 (8.68)	17.94 (8.42)	0.34	16	.74
CAMS^e^	37.24 (4.45)	37.18 (4.46)	0.07	16	.95
MSMR^f^	62.53 (5.40)	62.71 (7.02)	–0.11	16	.91
MSMR_IR^g^	20.71 (3.06)	20.00 (3.39)	0.8	16	.44
MSMR_CP^h^	22.18 (2.53)	22.41 (2.55)	–0.30	16	.77
MSMR_ER^i^	19.65 (2.37)	20.29 (3.02)	–1.11	16	.28
MSMR_HealthWellness	5.82 (1.81)	6.18 (1.98)	–0.88	16	.39
MSMR_HealthReserve	7.94 (1.30)	7.24 (2.25)	1.76	16	.10
MSMR_PsychRegulation	6.94 (1.95)	6.59 (1.73)	0.82	16	.42
MSMR_LifeSatisfaction	8.24 (0.97)	8.47 (0.94)	–0.78	16	.45
MSMR_GrowthCapacity	8.59 (0.80)	8.06 (1.43)	1.77	16	.10
MSMR_SelfEsteem	5.35 (2.40)	5.88 (1.90)	–0.80	16	.43
MSMR_SocialSecurity	6.47 (1.62)	6.65 (1.77)	–0.43	16	.68
MSMR_SocialFunction	6.47 (1.42)	6.06 (1.25)	1	16	.33
MSMR_AccSupport	6.71 (1.96)	7.59 (1.28)	–1.74	16	.10

^a^DASS-D: Depression, Anxiety and Stress Scale–depression.

^b^DASS-A: Depression, Anxiety and Stress Scale–anxiety.

^c^DASS-S: Depression, Anxiety and Stress Scale–stress.

^d^AAQ-2: Acceptance and Action Questionnaire–2.

^e^CAMS: Cognitive and Affective Mindfulness Scale.

^f^MSMR: Multi-System Model of Resilience.

^g^MSMR_IR: Multi-System Model of Resilience–internal resilience.

^h^MSMR_CP: Multi-System Model of Resilience–coping pursuits.

^i^MSMR_ER: Multi-System Model of Resilience–external resilience.

**Table 3:** Paired sample *t* test among intervention participants at baseline and 6 weeks postintervention.

Variable	Baseline (n=17), mean (SD)	6 weeks postintervention (n=17), mean (SD)	*t* score	*df*	*P* value
DASS-D^a^	6.47 (10.67)	3.88 (6.65)	1.14	16	.27
DASS-A^b^	8.71 (9.25)	6.71 (7.17)	0.80	16	.43
DASS-S^c^	10.59 (9.92)	5.06 (4.25)	2.60	16	.02
AAQ-2^d^	18.65 (8.68)	16.24 (7.05)	1.13	16	.28
CAMS^e^	37.24 (4.45)	36.76 (4.42)	0.49	16	.63
MSMR^f^	62.53 (5.40)	65.35 (5.93)	–3.33	16	.004
MSMR_IR^g^	20.71 (3.06)	20.88 (2.52)	–0.33	16	.75
MSMR_CP^h^	22.18 (2.53)	23.12 (2.37)	–1.59	16	.13
MSMR_ER^i^	19.65 (2.37)	21.35 (3.06)	–2.71	16	.02
MSMR_HealthWellness	5.82 (1.81)	5.94 (1.48)	–0.36	16	.73
MSMR_HealthReserve	7.94 (1.30)	8.00 (1.27)	–0.22	16	.83
MSMR_PsychRegulation	6.94 (1.95)	6.94 (1.39)	0	16	>.99
MSMR_LifeSatisfaction	8.24 (0.97)	8.59 (0.87)	–1.69	16	.11
MSMR_GrowthCapacity	8.59 (0.80)	8.23 (0.97)	1.46	16	.16
MSMR_SelfEsteem	5.35 (2.40)	6.29 (1.96)	–1.96	16	.07
MSMR_SocialSecurity	6.47 (1.62)	6.82 (1.51)	–1.24	16	.23
MSMR_SocialFunction	6.47 (1.42)	6.65 (1.58)	–0.34	16	.74
MSMR_AccSupport	6.71 (1.96)	7.88 (1.50)	–2.85	16	.01

^a^DASS-D: Depression, Anxiety and Stress Scale–depression.

^b^DASS-A: Depression, Anxiety and Stress Scale–anxiety.

^c^DASS-S: Depression, Anxiety and Stress Scale–stress.

^d^AAQ-2: Acceptance and Action Questionnaire–2.

^e^CAMS: Cognitive and Affective Mindfulness Scale.

^f^MSMR: Multi-System Model of Resilience.

^g^MSMR_IR: Multi-System Model of Resilience–internal resilience.

^h^MSMR_CP: Multi-System Model of Resilience–coping pursuits.

^i^MSMR_ER: Multi-System Model of Resilience–external resilience.

**Table 4:** Independent sample *t* test between study arms postintervention.

Variable	Intervention (n=17), mean (SD)	Control (n=12), mean (SD)	*t* score	*df*	*P* value
DASS-D^a^	6.94 (9.83)	9.50 (12.33)	–0.60	20.27	.56
DASS-A^b^	6.94 (5.57)	10.83 (8.96)	–1.33	16.96	.20
DASS-S^c^	7.76 (6.32)	10.33 (9.94)	–0.79	17.21	.44
AAQ-2^d^	17.94 (8.42)	18.00 (12.60)	–0.01	17.82	.99
CAMS^e^	37.18 (4.46)	32.92 (5.04)	2.35	21.94	.03
MSMR^f^	62.71 (7.02)	55.67 (12.31)	1.79	16.06	.09
MSMR_IR^g^	20.00 (3.39)	19.42 (4.21)	0.40	20.44	.70
MSMR_CP^h^	22.41 (2.55)	19.75 (5.75)	1.50	14.08	.16
MSMR_ER^i^	20.29 (3.02)	16.50 (3.61)	2.98	21.02	.007
MSMR_Health wellness	6.18 (1.98)	5.25 (2.09)	1.20	22.94	.24
MSMR_Health reserve	7.24 (2.25)	7.33 (1.37)	–0.15	26.56	.89
MSMR_Psychological regulation	6.59 (1.73)	6.83 (1.80)	–0.37	23.26	.72
MSMR_Life satisfaction	8.47 (0.94)	6.75 (2.34)	2.41	13.55	.03
MSMR_Growth capacity	8.06 (1.43)	6.83 (2.44)	1.56	16.34	.14
MSMR_Self esteem	5.88 (1.90)	6.17 (2.52)	–0.33	19.47	.74
MSMR_Social security	6.65 (1.77)	5.75 (2.01)	1.25	21.84	.23
MSMR_Social function	6.06 (1.25)	5.42 (1.78)	1.15	19.76	.26
MSMR_Accessible support	7.59 (1.28)	5.33 (2.35)	3.03	15.61	.008

^a^DASS-D: Depression, Anxiety and Stress Scale–depression.

^b^DASS-A: Depression, Anxiety and Stress Scale–anxiety.

^c^DASS-S: Depression, Anxiety and Stress Scale–stress.

^d^AAQ-2: Acceptance and Action Questionnaire–2.

^e^CAMS: Cognitive and Affective Mindfulness Scale.

^f^MSMR: Multi-System Model of Resilience.

^g^MSMR_IR: Multi-System Model of Resilience–internal resilience.

^h^MSMR_CP: Multi-System Model of Resilience–coping pursuits.

^i^MSMR_ER: Multi-System Model of Resilience–external resilience.

**Table 5:** Linear mixed effects models: effects of WE2CARE on outcomes among intervention participants.

Parameter	Coefficient (95% CI)	Standard error	*t* score	*P* value	Random effect (SD)
Intercept	7.88 (3.10 to 12.66)	2.42	3.26	.005	7.59
DASS_D^a^	–0.59 (–5.05 to 3.87)	2.21	–0.27	.79	6.46
Intercept	8.71 (5.08 to 12.33)	1.85	4.70	<.001	2.35
DASS_A^b^	–1.76 (–6.78 to 3.25)	2.49	–0.71	.49	7.26
Intercept	10.59 (6.64 to 14.53)	2.02	5.25	<.001	1.91
DASS_S^c^	–2.82 (–8.37 to 2.72)	2.78	–1.02	.32	8.09
Intercept	18.65 (14.56 to 22.74)	2.07	8.99	<.001	6.04
AAQ-2^d^	–0.71 (–4.89 to 3.47)	2.08	–0.34	.74	6.05
Intercept	37.24 (35.09 to 39.38)	1.08	34.45	<.001	3.67
CAMS^e^	–0.06 (–1.81 to 1.69)	0.87	–0.07	.95	2.53
Intercept	62.53 (59.54 to 65.52)	1.52	41.16	<.001	4.23
MSMR^f^	0.18 (–3.01 to 3.37)	1.58	0.11	.91	4.62
Intercept	20.71 (19.17 to 22.24)	0.78	26.44	<.001	1.95
MSMR_IR^g^	–0.71 (–2.48 to 1.07)	0.88	–0.80	.44	2.57
Intercept	22.18 (20.97 to 23.38)	0.62	35.99	<.001	1.05
MSMR_CP^h^	0.24 (–1.36 to 1.83)	0.79	0.30	.77	2.31
Intercept	19.65 (18.34 to 20.95)	0.66	29.87	<.001	2.12
MSMR_ER^i^	0.65 (–0.52 to 1.82)	0.58	1.11	.28	1.69
Intercept	5.82 (4.91 to 6.73)	0.28	30.53	<.001	0.77
MSMR_HealthWellness	0.35 (–0.46 to 1.16)	0.30	–1.77	.10	0.87
Intercept	7.94 (7.06 to 8.82)	0.52	10.21	<.001	0.99
MSMR_HealthReserve	–0.71 (–1.51 to 0.10)	0.66	0.80	.43	1.92
Intercept	6.94 (6.06 to 7.83)	0.41	15.72	<.001	1.19
MSMR_PsychRegulation	–0.35 (–1.22 to 0.51)	0.41	0.43	.68	1.21
Intercept	8.24 (7.78 to 8.69)	0.28	30.53	<.001	0.77
MSMR_LifeSatisfaction	0.24 (–0.38 to 0.85)	0.30	–1.77	.10	0.87
Intercept	8.59 (8.03 to 9.14)	0.28	30.53	<.001	0.77
MSMR_GrowthCapacity	–0.53 (–1.13 to 0.07)	0.30	–1.77	.10	0.87
Intercept	5.35 (4.33 to 6.38)	0.52	10.21	<.001	0.99
MSMR_SelfEsteem	0.53 (–0.80 to 1.86)	0.66	0.80	.43	1.92
Intercept	6.47 (5.66 to 7.28)	0.41	15.72	<.001	1.19
MSMR_SocialSecurity	0.18 (–0.66 to 1.01)	0.41	0.43	.68	1.21
Intercept	6.47 (5.84 to 7.11)	0.32	19.96	<.001	0.59
MSMR_SocialFunction	–0.41 (–1.24 to 0.42)	0.41	–1.00	.33	1.2
Intercept	6.71 (5.92 to 7.49)	0.40	16.71	<.001	0.75
MSMR_AccSupport	0.88 (–0.14 to 1.90)	0.51	1.74	.10	1.48

^a^DASS-D: Depression, Anxiety and Stress Scale–depression.

^b^DASS-A: Depression, Anxiety and Stress Scale–anxiety.

^c^DASS-S: Depression, Anxiety and Stress Scale–stress.

^d^AAQ-2: Acceptance and Action Questionnaire–2.

^e^CAMS: Cognitive and Affective Mindfulness Scale.

^f^MSMR: Multi-System Model of Resilience.

^g^MSMR_IR: Multi-System Model of Resilience–internal resilience.

^h^MSMR_CP: Multi-System Model of Resilience–coping pursuits.

^i^MSMR_ER: Multi-System Model of Resilience–external resilience.

Looking across the 2 arms of the study, even though the mean scores for DASS-D, DASS-A, and DASS-S decreased more among the intervention group than the control, these changes were not significant. Furthermore, there were no significant differences in overall MSMR-I scores between intervention and control participants. However, there were significant increases postintervention in mindful qualities (CAMS-R) and external resilience (MSMR-ER), particularly in life satisfaction and accessible support among the intervention group (37.18 vs 32.92, t_22_=2.35, *P*=.03; 20.29 vs 16.5, t_21_=2.98, *P*=.007; 8.47 vs 6.75, t_14_=2.41, *P*=.03; 7.59 vs 5.33, t_16_=.008, respectively).

For the linear mixed effects models (Tables 5-6), the following outcomes saw a greater amount of improvement among intervention participants than controls. Compared with control group, those in the intervention group experienced a decrease in anxiety (–1.76 vs –0.50) and stress level (–2.82 vs –1.5) and an improvement in mindful qualities (–0.06 vs –2.75) and external resilience, particularly with respect to accessible support (0.88 vs 0.42), life satisfaction (0.24 vs –0.17), social security (0.18 vs –0.33), and self-esteem (0.53 vs 0.5).

**Table 6:** Linear mixed effects models: effects of WE2CARE on outcomes among control participants.

Parameter	Coefficient (95% CI)	Standard error	*t* score	*P* value	Random effect (SD)
Intercept	12.00 (5.03 to 18.97)	3.49	3.44	.006	10.16
DASS-D^a^	–2.50 (–7.93 to 2.93)	2.66	–0.94	.37	6.53
Intercept	11.33 (6.06 to 16.60)	2.65	4.28	.001	7.13
DASS-A^b^	–0.50 (–5.31 to 4.31)	2.36	–0.21	.84	5.79
Intercept	11.83 (5.49 to 18.18)	3.17	3.73	.003	9.23
DASS-S^c^	–1.50 (–6.47 to 3.47)	2.44	–0.62	.55	5.97
Intercept	24.25 (16.47 to 32.03)	3.85	6.29	.001	12.48
AAQ-2^d^	–6.25 (–10.20 to –2.30)	1.94	–3.22	.008	4.75
Intercept	35.67 (32.86 to 38.47)	1.39	25.57	<.001	4.29
CAMS^e^	–2.75 (–4.59 to –0.91)	0.91	–3.04	.01	2.22
Intercept	52.92 (44.70 to 61.13)	4.07	13.00	<.001	13.08
MSMR^f^	2.75 (–1.64 to 7.14)	2.15	1.28	.23	5.28
Intercept	17.25 (14.36 to 20.14)	1.45	11.90	<.001	4.10
MSMR_IR^g^	2.17 (–0.25 to 4.58)	1.19	1.83	.09	2.91
Intercept	19.25 (15.63 to 22.87)	1.79	10.78	<.001	5.90
MSMR_CP^h^	0.50 (–1.06 to 2.06)	0.76	0.65	.53	1.87
Intercept	16.42 (14.04 to 18.80)	1.18	13.87	<.001	3.69
MSMR_ER^i^	0.08 (–1.41 to 1.58)	0.73	0.11	.91	1.80
Intercept	4.58 (3.37 to 5.79)	0.62	7.40	<.001	0.45
MSMR_HealthWellness	0.67 (–1.04 to 2.37)	0.86	0.78	.45	2.10
Intercept	6.50 (5.24 to 7.76)	0.64	10.18	<.001	1.60
MSMR_HealthReserve	0.83 (–0.44 to 2.11)	0.63	1.33	.21	1.53
Intercept	6.17 (5.00 to 7.34)	0.59	10.37	<.001	1.26
MSMR_PsychRegulation	0.67 (–0.69 to 2.02)	0.67	1.00	.34	1.63
Intercept	6.92 (5.57 to 8.27)	0.67	10.29	<.001	2.08
MSMR_LifeSatisfaction	–0.17 (–1.03 to 0.70)	0.42	–0.39	.70	1.04
Intercept	6.67 (5.29 to 8.04)	0.69	9.71	<.001	2.03
MSMR_GrowthCapacity	0.17 (–0.86 to 1.20)	0.51	0.33	.75	1.24
Intercept	5.67 (4.06 to 7.27)	0.79	7.14	<.001	2.59
MSMR_SelfEsteem	0.50 (–0.27 to 1.27)	0.38	1.32	.21	0.93
Intercept	5.42 (3.96 to 6.88)	0.74	7.36	<.001	1.91
MSMR_SocialSecurity	–0.33 (–1.07 to 1.74)	0.69	0.48	.64	1.69
Intercept	6.08 (5.04 to 7.12)	0.52	11.66	<.001	1.46
MSMR_SocialFunction	–0.67 (–1.55 to 0.21)	0.43	–1.54	.15	1.06
Intercept	4.92 (3.62 to 6.21)	0.66	7.50	<.001	1.55
MSMR_AccSupport	0.42 (–0.97 to 1.80)	0.68	0.61	.55	1.66

^a^DASS-D: Depression, Anxiety and Stress Scale–depression.

^b^DASS-A: Depression, Anxiety and Stress Scale–anxiety.

^c^DASS-S: Depression, Anxiety and Stress Scale–stress.

^d^AAQ-2: Acceptance and Action Questionnaire–2.

^e^CAMS: Cognitive and Affective Mindfulness Scale.

^f^MSMR: Multi-System Model of Resilience.

^g^MSMR_IR: Multi-System Model of Resilience–internal resilience.

^h^MSMR_CP: Multi-System Model of Resilience–coping pursuits.

^i^MSMR_ER: Multi-System Model of Resilience–external resilience.

### Self-Reported WE2CARE Impact

Table 7 displays the participants’ self-reported satisfaction with WE2CARE intervention. Significant differences in the evaluation of the WE2CARE program were observed between the intervention and the control group. Overall, those in the intervention group gained more knowledge, confidence (self-efficacy), and behavioral activation than those in the control group. Those in the intervention group reported gaining more knowledge about stigma, having a clear idea about their values and being able to take committed actions that are in line with those values, and being aware of and able to access health and social services in their community. Moreover, they reported improvement in accessing health information, practicing self-care, receiving peer support, and engaging in community activities.

**Table 7:** Evaluation of WE2CARE postintervention by study arm allocation.

Participant Feedback	ACT^a^ (n=17)	Control (n=12)
1. I have gained more knowledge about stigma.	4.47 (0.62)	2.83 (1.11)
2. I am clearer about my values (or what matters to me).	4.88 (0.33)	4.00 (0.60)
3. I am more able to act based on my values (or what matters to me).	4.82 (0.39)	4.00 (0.60)
4. I am more aware of health services in my community.	4.71 (0.47)	3.92 (1.00)
5. I am more aware about of social services in my community.	4.65 (0.61)	3.75 (1.22)
6. I am more able to access services to support my health.	4.76 (0.44)	3.58 (0.90)
7. I am more able to access information to support my health.	4.82 (0.39)	3.67 (0.98)
8. I am more able to keep my prescribed treatments.	4.29 (1.10)	3.75 (0.75)
9. I am more able to practice self-care to support my health.	4.82 (0.39)	4.00 (0.74)
10. I have gained more peer support.	4.76 (0.44)	3.83 (0.94)
11. I am more able to provide peer support.	4.47 (0.62)	3.83 (0.94)
12. I have gained in more community activities that support my health and well-being.	4.59 (0.71)	3.83 (0.83)
Total	56.06 (3.25)	45.00 (8.02)

^a^ACT: acceptance and commitment therapy.

## Discussion

### Principal Findings

Our pilot study is among the first to use an online ACT intervention to address psychological distress among temporary migrant live-in caregivers. Our findings suggest that this intervention highly motivated participants to engage collectively in building social support networks and to some extent improved their mental health and resiliency.

The study participant sociodemographic characteristics and their living and working conditions were comparable to earlier studies [3,8,9,17,21]. Most reported working long hours and earning close to or below the Canadian low-income after-tax cutoff point. They were also concerned about the lack of privacy and inability to have control over their living/working conditions. About two-thirds reported being stressed and a fifth rated their mental health as fair/poor since arrival to Canada. The result of this study supports the healthy immigrant effect (ie, migrant workers arrive healthy as indicated during predeparture medical screening); however, their health status declines during their stay in Canada [21,44-46]. Although the participants in our study reported being healthy at the time of entry to Canada, a requirement for receiving a temporary work permit in Canada, they expressed a gradual decline in their physical and mental health. It is also interesting that despite having access to Ontario Health Insurance Plan, none of the participants had been tested for human papillomavirus, a known risk factor for cervical cancer. This highlights that the health of temporary female migrant workers particularly in the area of cervical cancer screening is ignored in our Canadian health care system and corroborates earlier findings about cancer screening disparities among vulnerable immigrant women [47-51].

This pilot waitlist-controlled trial found preliminary support for psychosocial improvements for the treatment group in comparison with the control group. A steady reduction in the levels of depression, anxiety, and stress were noted among the intervention group compared with control group even though these changes, except for stress level, were not statistically significant. The pattern of reduction is consistent with earlier studies exploring the impact of ACT on psychological distress among clinical and nonclinical population [27-29,52,53]. The inability to show significance may either be due to the small sample size or to the fact that our pilot study coincided with the COVID-19 crisis, which caused more psychosocial distress for our participants who were anxious about their own health and their loved ones back home. They may also have experienced COVID-19–related racism, which could have resulted in poorer mental health. Furthermore, during 2019, the Caregiver Program was further revised, and new conditions and programs were introduced. The two pilot programs that the Canadian government introduced in 2014 to replace Live-in Caregiver Program, the Caring for Children and Caring for People With High Medical Needs pilots, were replaced with two new pilots, the Home Child Care Provider and Home Support Worker pilots. Minimum language requirements and education credentials that restricted direct eligibility for permanent residency stayed unchanged. These changes caused further confusion, anger, and stress for temporary migrant caregivers as they had to again be reshuffled in the list of those waiting for their permanent residency. These conditions may have negatively impacted the mental health of participants. However, despite these conditions, the treatment group’s mental health still showed improvement postintervention and at 6-week follow-up.

Our pilot data also showed a significant worsening of mindfulness in the control group compared with the intervention group. This indicates that our intervention promoted participant uptake and use of mindfulness strategies. Considering this study took place during COVID-19, which increased mental distress, our data show that our intervention group may have benefited from the use of mindfulness exercises and teaching provided in our weekly modules compared with the control group, which had no access to this intervention. Empirical evidence suggests that mindfulness is associated with attention to and continuous engagement with both positive and negative experiences rather than avoidance of internal negative experiences [54,55]. Acceptance of one’s life experiences is a core ACT process that allows people to accept negative thoughts and feelings without being characterized by them, which in turn promotes their self-esteem and resiliency.

It is reported that ACT promotes psychological flexibility using experiential and attentional exercises (mindfulness), clarifications of values, and committed actions directed by values [22]. Our study found an increase in psychological flexibility among the control compared with the intervention group. This is because the participants’ mean score was higher in the control group compared with the intervention group prior to start of the intervention (23.75 vs 18.65) and dropped postintervention (18 vs 17.94), In other words, there was a bigger drop in mean scores in the control group because they were less flexible at the start of the study. This may be due to selection bias, as we used nonprobability sampling strategies to recruit our sample, as well as our small sample size. Future study with a larger sample size is needed to examine the impact of ACT on psychological flexibility through online delivery.

Furthermore, we found a significant increase in external resiliency among participants in the intervention group compared with the control group specifically across the dimensions of life satisfaction and accessible support. Our weekly videoconferencing provided an opportunity for our participants to connect with their peers and engage collectively in building social support networks. The weekly videoconferencing provided a vehicle to facilitate the building of a virtual community of mutual support that continued beyond the project. Some of the participants continued connecting through social media like WhatsApp.

The results from the evaluation survey component where participants were asked to comment on the utility of the intervention further support the positive influence WE2CARE had on participants. Compared with the control group, those in the intervention group self-reported an increase in knowledge about stigma, gained more peer support, and participated in more community activities that supported their health and well-being. This demonstrates how ACT equipped participants with the ability to expand their social network that supports their health. These findings suggest that participants made improvements in their resiliency and coping after receiving WE2CARE.

Our study offers numerous implications for practice: (1) evidence-informed online interventions enhance participation access and implementation feasibility, (2) web-based interventions can be effective in promoting mental health, (3) the combined use of individual self-directed e-learning and group videoconferencing allows peer connection and reduce social isolation, and (4) online group videoconferencing offers opportunities for marginalized groups, like temporary foreign workers, to get connected and engage in social action to challenge existing exploitative policies and practices to achieve equity.

### Limitations

There are some limitations to this study that should be considered when reviewing the results. First, the small sample size limits our ability to generalize findings to the larger community of migrant live-in caregivers. However, the goal of this study is not to generalize to the larger community of migrant live-in caregivers but rather to explore the feasibility of an internet-based psychological intervention in promoting caregiver mental well-being. Furthermore, due to the nature of a pilot study, having a small sample size is acceptable considering the paucity of information surrounding not only the use of ACT in reducing psychological distress but also internet delivery of the intervention for migrant live-in caregivers. Second, the study relied on self-report measurements that are prone to biases in this type of assessment (eg, social desirability, environmental biases like fatigue or privacy). A combination of self-report measures with physiological measures may deliver further insight. However, the unique living and working conditions of our target population (eg, long working hours, limited free time, fear of deportation due to health issues) in addition to COVID-19 public health restrictions made the use of self-report measures a viable option. Third, considering Caregiver Program reforms in November 2014, some migrant caregivers may be living outside their employers’ home. Except for 2 participants in our study, the rest lived with their employers. Hence, it would be important for future studies to explore the efficacy of ACT in reducing psychological distress among those migrant caregivers who live outside their place of employment.

### Conclusion

WE2CARE is among the first studies to explore the effectiveness of ACT in addressing mental health challenges among temporary migrant live-in caregivers. Our pilot data provided preliminary results on the efficacy of ACT in reducing mental health distress and promoting self-care. The results help to inform the development of culturally safe web-based interactive programs to increase access to individual psychological support among socially isolated and marginalized groups, promote the establishment of peer social networks and supportive environments, and promote collective engagement toward advancing social change. A large-scale study is warranted to confirm the preliminary results obtained in this study. There is a great potential for adapting WE2CARE for use with other temporary foreign workers like seasonal migrant farm workers across Ontario, other Canadian provinces, and internationally.

## Appendix

Multimedia Appendix 1 Consort-EHEALTH V1.6.

## Abbreviations

AAQ-2: Acceptance and Action Questionnaire–2
ACT: Acceptance and Commitment Therapy
CAMS-R: Cognitive and Affective Mindfulness Scale
DASS-21: Depression, Anxiety and Stress Scale
DASS-A: Depression, Anxiety and Stress Scale–anxiety
DASS-D: Depression, Anxiety and Stress Scale–depression
DASS-S: Depression, Anxiety and Stress Scale–stress
MSMR-CP: Multi-System Model of Resilience–coping pursuits
MSMR-ER: Multi-System Model of Resilience–external resilience
MSMR-I: Multi-System Model of Resilience
MSMR-IR: Multi-System Model of Resilience–internal resilience
WE2CARE: Women Empowerment—Caregiver Acceptance and Resilience E-Learning
