# Supplementary material for: Effects of Acceptance and Commitment Therapy (ACT) on Mental Health and Resiliency of Migrant Live-in Caregivers in Canada: Pilot Randomized Wait List Controlled Trial
Source: JMIR Form Res. 2022 Jan 27;6(1):e32136. doi: 10.2196/32136 (PMC9090443; doi:10.2196/32136)
Supplement: Multimedia Appendix 1 [file formative_v6i1e32136_fig.pdf]

CONSORT-EHEALTH Checklist V1.6.2 Report	Manuscript Number	32136
**(based on CONSORT-EHEALTH V1.6), available at [http://tinyurl.com/consort-ehealth-v1-6].**		
**Date completed**		
1/24/2022 15:08:03		
**by**		
Mandana Vahabi		
Effects of Acceptance and Commitment Therapy (ACT) on Mental Health and Resiliency of Migrant Live-in Caregivers in Canada:Pilot Randomized Wait List Controlled Trial		
TITLE		
**1a-i) Identify the mode of delivery in the title**		
Yes--"Given that live-in caregivers work long hours and have extremely limited free time and restricted social support, a web-based approach is the best-suited medium for the delivery of self-help psychological treatment in this population"; Each week, participants were invited to complete an online self-directed, interactiveexperiential session on ACT strategies (approximately 1 hour to complete) and attend a 1.5-hour online live videoconference."		
**1a-ii) Non-web-based components or important co-interventions in title**		
No-- The study was based on the target population busy schedule. Web-based intervention was the only mode of delivery that participants were eager to use.		
**1a-iii) Primary condition or target group in the title**		
Yes--"Temporary migrant live-in caregivers constitute a vulnerable stream of temporary foreign workers in Canada. This is because the majority are racialized women from the Global South, the gendered nature of caregiving work has historically been undervalued, and their working and living spheres are intertwined which makes application of labor laws and surveillance almost impossible. Their invisible position in the fabric of Canadian society along with their precarious employment and immigration status place their mental health at jeopardy. There is a paucity of research about psychological support for this population."		
ABSTRACT		
**1b-i) Key features/functionalities/components of the intervention and comparator in the METHODS section of the ABSTRACT**		
Yes, please see the abstract		
**1b-ii) Level of human involvement in the METHODS section of the ABSTRACT**		
to some degree as was allowed with the limited space		
**1b-iii) Open vs. closed, web-based (self-assessment) vs. face-to-face assessments in the METHODS section of the ABSTRACT**		
Yes, captured in the abstract		
**1b-iv) RESULTS section in abstract must contain use data**		
Yes, Fig 1 shows the number of participants recruited for this stidy		
**1b-v) CONCLUSIONS/DISCUSSION in abstract for negative trials**		
Yes, it is captured please see the conclusion section in our abstract		
INTRODUCTION		
**2a-i) Problem and the type of system/solution**		
Yes, see the paper introduction section		
**2a-ii) Scientific background, rationale: What is known about the (type of) system**		
Yes, See the introduction section of the paper		
**Does your paper address CONSORT subitem 2b?**		
Yes, objectives and hypothesis are captured. See Introduction		
METHODS		
**3a) CONSORT: Description of trial design (such as parallel, factorial) including allocation ratio**		
a randomized controlled wait list design		
**3b) CONSORT: Important changes to methods after trial commencement (such as eligibility criteria), with reasons**		
No changes were made to methods after trial commencement		
**3b-i) Bug fixes, Downtimes, Content Changes**		
No, since we did niot have any issue in this area		
**4a) CONSORT: Eligibility criteria for participants**		
Eligible participants meeting the study inclusion criteria: (1) self-identified as female aged 18 years or older,; (2) were residing in the Greater Toronto Area (GTA),; (3) were working on a temporary work permit as a live-in caregivers,; (4) were able to speak and read English,; (5) had internet access,, and (6) were able to take part in the 6-week intervention.		
**4a-i) Computer / Internet literacy**		
All participants have experienced using computer and internet as live in acre givers		
**4a-ii) Open vs. closed, web-based vs. face-to-face assessments:**		
Participants were recruited by two 2 community champions (trusted members of live-in caregivers' community) and snowball technique.		
**4a-iii) Information giving during recruitment**		
It only states that our protocol received ethics approval		
**4b) CONSORT: Settings and locations where the data were collected**		
All information were collected virtually		
**4b-i) Report if outcomes were (self-)assessed through online questionnaires**		
Yes, please see the data collection and analyses		
**4b-ii) Report how institutional affiliations are displayed**		
Yes, it clearly states in consent form.		
**5) CONSORT: Describe the interventions for each group with sufficient details to allow replication, including how and when they were actually administered**		
**5-i) Mention names, credential, affiliations of the developers, sponsors, and owners**		
Please see intervention section		
**5-ii) Describe the history/development process**		
No, we do not discuss this		
**5-iii) Revisions and updating**		
No changes were made to proposed intervention		
**5-iv) Quality assurance methods**		
There has not been major differences in implemetation phase		
**5-v) Ensure replicability by publishing the source code, and/or providing screenshots/screen-capture video, and/or providing flowcharts of the algorithms used**		
No, but we will be open to providing aggregated data to resaerchers who may be interested to replicate this study		
**5-vi) Digital preservation**		
Our budget was not enough to pay for the cost after the completion of the study		
**5-vii) Access**		
See methods section of paper		
**5-viii) Mode of delivery, features/functionalities/components of the intervention and comparator, and the theoretical framework**		
See methods section of paper		
**5-ix) Describe use parameters**		
See methods section- "Details of the study protocol have been reported elsewhere-http://preprints.jmir.org/preprint/31211 DOI: 10.2196/preprints.31211		
**5-x) Clarify the level of human involvement**		
Only research coordinator/assistand is reported		
**5-xi) Report any prompts/reminders used**		
Not captured in the manuscript		
**5-xii) Describe any co-interventions (incl. training/support)**		
Partially about the qualification of facilitators		
**6a) CONSORT: Completely defined pre-specified primary and secondary outcome measures, including how and when they were assessed**		

Data were captured through self-report instruments administered online at pre-, post-, and 6- weeks post-intervention. The pre-intervention survey included socio-demographic and health- related questions which that wereas identified as important in previous research with temporary migrant workers [14,16]. The standardized scales administered at pre-, post-, and 6-week follow-up included: (1i) Depression, Anxiety and Stress Scale (DASS-21)— – a set of three 3 self-report scales (21 items) designed to measure the emotional states of depression (DASS-D), anxiety (DASS-A) and stress (DASS-S); Cronbach's alpha values of 0.81, 0.89 and 0.78 for the subscales of depressive, anxiety and stress respectively. (2ii) Acceptance and Action Questionnaire–-2 (AAQ-2) — – a 7-item scale specifically designed to measure the impact of ACT core process conceptualized as psychological flexibility; (3iii) Cognitive and Affective Mindfulness Scale (CAMS-R)— - a 12-item measure designed to capture a broad conceptualization of mindfulness not specific to any particular type of meditation training; and the (4iv) Multi-System Model of Resilience (MSMR-I), consistings of three 3 subscales: internal resilience (MSMR-IR), coping pursuits (MSMR-CP), and external resilience (MSMR-ER). Each subscale contains 9 self-reported items and indicates where the barriers to one's resilience lie. These scales have shown good psychometric properties including internal consistency, test-retest reliability, and validity. For instance, the depressive, anxiety, and stress subscales in DASS have been have found to have excellent Cronbach's alpha values of .81, .89, and .78, respectively., AAQ-2 was reported to have good internal consistency (α = 0.88) and good test retest reliability over 3 and 12 months at .81 and .79, respectively. CAMS-R was reported to have good Cronbach alpha (.67) and good convergent validity that is supported by its negative relationship to the DASS-21 is negatively correlated to DASS (-.(–.28). MSMR-I also showed excellent internal consistency with Cronbach's alphaα of .90 and high test-retest reliability .84		
**6a-i) Online questionnaires: describe if they were validated for online use and apply CHERRIES items to describe how the questionnaires were designed/deployed**		
All questionnaire were validated and reliable		
**6a-ii) Describe whether and how "use" (including intensity of use/dosage) was defined/measured/monitored**		
See the intervention sectio		
**6a-iii) Describe whether, how, and when qualitative feedback from participants was obtained**		
This is not included here but we are working on another paper which reports the qualitative feedback		
**6b) CONSORT: Any changes to trial outcomes after the trial commenced, with reasons**		
All information were collected virtually		
**7a) CONSORT: How sample size was determined**		
**7a-i) Describe whether and how expected attrition was taken into account when calculating the sample size**		
This is a pilot study so sample size calculation was done		
**7b) CONSORT: When applicable, explanation of any interim analyses and stopping guidelines**		
Data were captured through self-report instruments administered online at pre-, post-, and 6- weeks post-intervention. The pre-intervention survey included socio-demographic and health- related questions which that wereas identified as important in previous research with temporary migrant workers [14,16]. The standardized scales administered at pre-, post-, and 6-week follow-up included: (1i) Depression, Anxiety and Stress Scale (DASS-21)— – a set of three 3 self-report scales (21 items) designed to measure the emotional states of depression (DASS-D), anxiety (DASS-A) and stress (DASS-S); Cronbach's alpha values of 0.81, 0.89 and 0.78 for the subscales of depressive, anxiety and stress respectively. (2ii) Acceptance and Action Questionnaire–-2 (AAQ-2) — – a 7-item scale specifically designed to measure the impact of ACT core process conceptualized as psychological flexibility; (3iii) Cognitive and Affective Mindfulness Scale (CAMS-R)— - a 12-item measure designed to capture a broad conceptualization of mindfulness not specific to any particular type of meditation training; and the (4iv) Multi-System Model of Resilience (MSMR-I), consistings of three 3 subscales: internal resilience (MSMR-IR), coping pursuits (MSMR-CP), and external resilience (MSMR-ER). Each subscale contains 9 self-reported items and indicates where the barriers to one's resilience lie. These scales have shown good psychometric properties including internal consistency, test-retest reliability, and validity. For instance, the depressive, anxiety, and stress subscales in DASS have been have found to have excellent Cronbach's alpha values of .81, .89, and .78, respectively., AAQ-2 was reported to have good internal consistency (α = 0.88) and good test retest reliability over 3 and 12 months at .81 and .79, respectively. CAMS-R was reported to have good Cronbach alpha (.67) and good convergent validity that is supported by its negative relationship to the DASS-21 is negatively correlated to DASS (-.(–.28). MSMR-I also showed excellent internal consistency with Cronbach's alphaα of .90 and high test-retest reliability .84		
**8a) CONSORT: Method used to generate the random allocation sequence**		
Used a computer software program (Excel microsoft) that generated the random sequence.		
**8b) CONSORT: Type of randomisation; details of any restriction (such as blocking and block size)**		
Simple randomization was used		
**9) CONSORT: Mechanism used to implement the random allocation sequence (such as sequentially numbered containers), describing any steps taken to conceal the sequence until interventions were assigned**		
No, the paper does not discuss how but states that participants were randomly assigned to intervention and control group. See Methods section		
**10) CONSORT: Who generated the random allocation sequence, who enrolled participants, and who assigned participants to interventions**		
We did not use random selection but rather relied on puposive sampling due to the fact that there is no sampling frame for live in care givers currently. We did discuss recruitment process and mentioned that particpants were allocated randomly to intervention and control group		
**11a) CONSORT: Blinding - If done, who was blinded after assignment to interventions (for example, participants, care providers, those assessing outcomes) and how**		
**11a-i) Specify who was blinded, and who wasn't**		
it is not possible to blind the participants		
**11a-ii) Discuss e.g., whether participants knew which intervention was the "intervention of interest" and which one was the "comparator"**		
There was only one intervention which was first provided to the intervention group. After the completion of the study and collection of post-test results. The intervention was offered to control group		
**11b) CONSORT: If relevant, description of the similarity of interventions**		
This is not relevant to our intervention		
**12a) CONSORT: Statistical methods used to compare groups for primary and secondary outcomes**		
Yes, Please see anlyses section of paper		
**12a-i) Imputation techniques to deal with attrition / missing values**		
NO, we excluded partial attendes		
**12b) CONSORT: Methods for additional analyses, such as subgroup analyses and adjusted analyses**		
Please see the analysis section		
RESULTS		
**13a) CONSORT: For each group, the numbers of participants who were randomly assigned, received intended treatment, and were analysed for the primary outcome**		
Yes, See the analyses reported in the paper		
**13b) CONSORT: For each group, losses and exclusions after randomisation, together with reasons**		
Yes, See methods section		
**13b-i) Attrition diagram**		
The explanation is provided in text under Methods section : participants and recruitment. There is also a figure that shows this		
**14a) CONSORT: Dates defining the periods of recruitment and follow-up**		
No provided in the paper.		
**14a-i) Indicate if critical "secular events" fell into the study period**		
No secular event occurred		
**14b) CONSORT: Why the trial ended or was stopped (early)**		
Our study ended after our data completion as usual practice.		
**15) CONSORT: A table showing baseline demographic and clinical characteristics for each group**		
Yes, please see Results section		
**15-i) Report demographics associated with digital divide issues**		
Please see Results section		
**16a) CONSORT: For each group, number of participants (denominator) included in each analysis and whether the analysis was by original assigned groups**		
**16-i) Report multiple "denominators" and provide definitions**		
please see Results section		
**16-ii) Primary analysis should be intent-to-treat**		
Not relevant --This is a pilot study		
**17a) CONSORT: For each primary and secondary outcome, results for each group, and the estimated effect size and its precision (such as 95% confidence interval)**		
Not relevant --This is a pilot study		
**17a-i) Presentation of process outcomes such as metrics of use and intensity of use**		
Not the focus of our pilot study		
**17b) CONSORT: For binary outcomes, presentation of both absolute and relative effect sizes is recommended**		
Not relevant --This is a pilot study		
**18) CONSORT: Results of any other analyses performed, including subgroup analyses and adjusted analyses, distinguishing pre-specified from exploratory**		
Please see the results section		

**18-i) Subgroup analysis of comparing only users**		
Please see teh results section of the paper		
**19) CONSORT: All important harms or unintended effects in each group**		
Not applicable to pur study		
**19-i) Include privacy breaches, technical problems**		
Included in the online consent form		
**19-ii) Include qualitative feedback from participants or observations from staff/researchers**		
We intend to publish another paper specifically on qualitaive comments which is currently underdevelopment		
DISCUSSION		
**20) CONSORT: Trial limitations, addressing sources of potential bias, imprecision, multiplicity of analyses**		
**20-i) Typical limitations in ehealth trials**		
This is a pilot study so the limitations included other issues. Plesae see Limitations section of the paper		
**21) CONSORT: Generalisability (external validity, applicability) of the trial findings**		
**21-i) Generalizability to other populations**		
Plesae see the limitaions section "the small sample size limits our ability to generalize findings to the larger community of migrant live-in caregivers."		
**21-ii) Discuss if there were elements in the RCT that would be different in a routine application setting**		
Not relevant to the study purpose		
**22) CONSORT: Interpretation consistent with results, balancing benefits and harms, and considering other relevant evidence**		
**22-i) Restate study questions and summarize the answers suggested by the data, starting with primary outcomes and process outcomes (use)**		
Plesae see the discussuon section		
**22-ii) Highlight unanswered new questions, suggest future research**		
The paper does offer future studies to explore the efficacy of ACT in reducing psychological distress among those migrant caregivers who live outside their place of employment.		
Other information		
**23) CONSORT: Registration number and name of trial registry**		
Not applicable for this study		
**24) CONSORT: Where the full trial protocol can be accessed, if available**		
The study protocol is published at http://preprints.jmir.org/preprint/31211 DOI: 10.2196/preprints.31211))		
**25) CONSORT: Sources of funding and other support (such as supply of drugs), role of funders**		
Yes, it include the funding source		
**X26-i) Comment on ethics committee approval**		
The study protocol received ethical approval from the rResearch eEthics rReview bBoards at the affiliated universities . Those included Ryerson University (REB 2019-036) and University of Toronto (RIS37623).		
**x26-ii) Outline informed consent procedures**		
NO this is not included in the paper but All consents were obtained online		
**X26-iii) Safety and security procedures**		
NO this is not included in the paper but All required measures were placed to ensure safety and security procedures		
**X27-i) State the relation of the study team towards the system being evaluated**		
Not really understood the question		
